# The influence of metformin treatment on the circulating proteome

**DOI:** 10.1016/j.ebiom.2025.105859

**Published:** 2025-07-19

**Authors:** Ben Connolly, Laura McCreight, Roderick C. Slieker, Khaled F. Bedair, Louise Donnelly, Juliette A. de Klerk, Joline W.J. Beulens, Petra J.M. Elders, Göran Bergström, Mun-Gwan Hong, Robert W. Koivula, Paul W. Franks, Jochen M. Schwenk, Anders Gummesson, Ewan R. Pearson, Leen M. ‘t Hart, IMI-DIRECT, Moustafa Abdalla, Jonathan Adam, Jerzy Adamski, Kofi Adragni, Rosa Lundbye L. Allesøe, Kristine H. Allin, Anna A. Artati, Manimozhiyan Arumugam, Naeimeh Atabaki Pasdar, Tania Baltauss, Karina Banasik, Anna Barnett, Patrick Baum, Jimmy D. Bell, Susanna Bianzano, Roberto Bizzotto, Amelie Bonnefond, Caroline Anna A. Brorsson, Andrew A. Brown, Søren Brunak, Louise Cabrelli, Robert Caiazzo, Henna Cederberg, Elizaveta Chabanova, Marc Clos-Garcia, Matilda Dale, David Davtian, Adem Y. Dawed, Federico De Masi, Nathalie de Preville, Koen F. Dekkers, Harshal A. Deshmukh, Christiane Dings, Avirup Dutta, Beate Ehrhardt, Line Engelbrechtsen, Rebeca Eriksen, Yong Fan, Juan Fernandez, Jorge Ferrer, Hugo Fitipaldi, Ian M. Forgie, Annemette Forman, Francesca Frau, Philippe Froguel, Gary Frost, Johann Gassenhuber, Giuseppe (Nick) N. Giordano, Toni Giorgino, Stephen Gough, Harald Grallert, Rolf Grempler, Lenka Groeneveld, Leif Groop, Valborg Gudmundsdóttir, Ramneek Gupta, Mark Haid, Torben Hansen, Tue H. Hansen, Andrew T. Hattersley, Ragna Haussler, Alison J. Heggie, Anita M. Hennige, Anita V. Hill, Reinhard W. Holl, Michelle Hudson, Bernd Jablonka, Ulrik Plesner Jacobsen, Christopher Jennison, Joachim Johansen, Angus G. Jones, Tugce Karaderi, Jane Kaye, Gwen Kennedy, Maria Klintenberg, Robert W. Koivula, Tarja Kokkola, Anitra D. Koopman, Azra Kurbasic, Teemu Kuulasmaa, Markku Laakso, Thorsten Lehr, Heather Loftus, Agnete Troen T. Lundgaard, Liwei Lyu, Anubha Mahajan, Andrea Mari, Gianluca Mazzoni, Mark I. McCarthy, Timothy J. McDonald, Donna McEvoy, Nicky McRobert, Ian McVittie, Miranda Mourby, Petra Musholt, Pascal Mutie, Rachel Nice, Claudia Nicolay, Giel Nijpels, Birgitte Nilsson, Colin N. Palmer, Francois Pattou, Imre Pavo, Helle K. Pedersen, Oluf Pedersen, Mandy H. Perry, Hugo Pomares-Millan, Cornelia P. Prehn, Anna Ramisch, Simon Rasmussen, Violeta Raverdi, Martin Ridderstråle, Neil Robertson, Marianne Rodriquez, Hartmut Ruetten, Femke Rutters, Peter Sackett, Nina Scherer, Nisha Shah, Sapna Sharma, Iryna Sihinevich, Nadja B. Sondertoft, Hans-Henrik Staerfeldt, Birgit Steckel-Hamann, Harriet Teare, Cecilia Engel E. Thomas, Elizabeth Louise L. Thomas, Melissa K. Thomas, Henrik S. Thomsen, Barbara Thorand, Claire E. Thorne, Joachim Tillner, Konstantinos D. Tsirigos, Andrea Tura, Mathias Uhlen, Sabine van Oort, Jagadish Vangipurapu, Helene Verkindt, Henrik Vestergaard, Ana Viñuela, Josef K. Vogt, Peter W. Wad Sackett, Mark Walker, Agata Wesolowska-Andersen, Brandon Whitcher, Margaret W. White, IMI-RHAPSODY, Alexander Efanov, Giuseppe N. Giordano, Gerard A. Bouland, Frédéric Burdet, Iulian Dragan, Andreas Festa, Michael K. Hansen, Dmitry Kuznetsov, Florence Mehl, Diana Marek, Imre Pavo, Kevin Duffin, Samreen K. Syed, Janice L. Shaw, Over Cabrera, Timothy J. Pullen, Bernard Thorens, Mark Ibberson, Guy A. Rutter

**Affiliations:** a Division of Population Health & Genomics, School of Medicine, University of Dundee, UK; b Department of Cell and Chemical Biology, Leiden University Medical Center, Leiden, the Netherlands; c Department of Internal Medicine (Nephrology), Leiden University Medical Center, Leiden, the Netherlands; d Department of Epidemiology and Data Sciences, Amsterdam University Medical Center, Location Vrije Universiteit Amsterdam, Amsterdam, the Netherlands; e Amsterdam Cardiovascular Sciences Research Institutes, Amsterdam Public Health, Amsterdam, the Netherlands; f Health Behaviors & Chronic Diseases Research Program & Personalised Medicine Research Program Amsterdam Public Health, Amsterdam, the Netherlands; g Department of General Practice Medicine, Amsterdam UMC, Location Vrije Universiteit, Amsterdam, the Netherlands; h Region Västra Götaland, Sahlgrenska University Hospital, Department of Clinical Physiology, Gothenburg, Sweden; i Department of Molecular and Clinical Medicine, Institute of Medicine, Sahlgrenska Academy, University of Gothenburg, Gothenburg, Sweden; j Department of Protein Science, SciLifeLab, KTH Royal Institute of Technology, Stockholm, Sweden; k Oxford Centre for Diabetes, Endocrinology and Metabolism, Radcliffe Department of Medicine, University of Oxford, Oxford, UK; l Genetic and Molecular Epidemiology Unit, Lund University Diabetes Centre, Department of Clinical Sciences, Lund University, Malmo, Sweden; m Precision Health University Research Institute, Queen Mary University of London, London, UK; n Region Västra Götaland, Sahlgrenska University Hospital, Department of Clinical Genetics and Genomics, Gothenburg, Sweden; o Department of Biomedical Data Sciences, Section Molecular Epidemiology, Leiden University Medical Center, Leiden, the Netherlands

**Keywords:** Metformin, Proteomics, Type 2 diabetes, Biomarker

## Abstract

**Background:**

Metformin is one of the most used drugs worldwide. Given the increasing use of proteomics in trials, bioresources, and clinics, it is crucial to understand the influence of metformin on the levels of the circulating proteome.

**Methods:**

We analysed a combined longitudinal proteomics dataset from the IMPOCT, RAMP and S3WP-T2D clinical trials in 98 participants before and after metformin exposure. This discovery analysis contained 372 proteins measured by proximity extension assays (Olink). We followed up experiment–wise statistically significant findings in two cross-sectional cohorts of people with type 2 diabetes comparing metformin treated and untreated individuals: IMI-DIRECT (784 participants, 372 proteins, Olink) and IMI-RHAPSODY (1175 participants, 1195 proteins, SomaLogic).

**Findings:**

Overall, 23 protein analytes were robustly associated with exposure to metformin in the discovery and replication. This includes 11 protein-metformin associations that replicated in both replication sets and platforms (REG4, GDF15, REG1A, t-PA, TFF3, CDH5, CNTN1, OMD, NOTCH3, THBS4 and CD93), with the remaining 12 protein-metformin associations replicated using the Olink platform (EPCAM, SPINK1, SAA-4, COMP, ITGB2, ADGRG2, FAM3C, MERTK, COL1A1, HAOX1, VCAN, TIMD4) but not measured on the SomaLogic platform. Gene-set enrichment analysis revealed that the metformin exposure was associated with intestinal associated proteins.

**Interpretation:**

These data highlight the need to account for exposure to metformin, and potentially other drugs, in proteomic studies and where protein biomarkers are used for clinical care.

**Funding:**

10.13039/501100010767Innovative Medicines Initiative Joint Undertaking 2, under grant agreement no. 115881 (RHAPSODY) and the 10.13039/501100010767Innovative Medicines Initiative Joint Undertaking under grant agreement no. 115317 (DIRECT), resources of which are composed of financial contribution from the European Union's Seventh Framework Programme (FP7/2007-2013) and 10.13039/100013322EFPIA companies in kind contribution as well as the Swiss State Secretariat for Education Research' and Innovation (SERI), under contract no. 16.0097 (RHAPSODY).

**Research in context**

**Evidence before this study**

Metformin is one of the most used drugs worldwide. Quantifying the effects of metformin on the circulating proteome is important for both enhanced understanding of the drug's mechanisms of action, as well as to help address the confounding role metformin may play in studies of protein-disease associations. There are limited systematic proteomic studies of metformin exposure, and these have largely been targeted using small panels of proteins or smaller, isolated sample sets.

**Added value of this study**

To better understand how metformin alters the circulating proteome, here we extend prior analyses in a comparatively large sample of participants from six different longitudinal studies, with repeat sampling before and after metformin initiation, and cross-sectional studies using two commonly used proteomic panels. We show that twenty-three proteins are robustly associated with metformin treatment.

**Implications of all the available evidence**

These data highlight the need to account for exposure to metformin, and potentially other drugs, in proteomic studies and where protein biomarkers are used for clinical care.

## Introduction

In recent years, there has been an increase in our ability to measure circulating plasma proteins at scale—measuring thousands of analytes in tens of thousands of people.^1^ This has helped increase biological understanding of disease as well as the potential use of proteomic panels for diagnosis, risk prediction and disease monitoring.^2^ However, like most other biomarkers, epidemiological associations between proteins and disease are prone to confounding. To ensure clinically robust, reproducible findings it is critical to account for this possibility in protein-disease association studies. Of the many potential confounders, drug treatment is an especially important example in disease cohorts owing to the physiological potency of medications.

Metformin has been used to treat diabetes for many decades, and today more than 200 million people are treated with the drug globally, yet its mechanisms of action remain poorly understood.^3^ Therefore, quantifying the effects of metformin on the circulating proteome is important for both enhanced understanding of the drug's mechanisms of action, as well as to help address the confounding role metformin may play in studies of protein-disease associations.

There are limited systematic proteomic studies of metformin exposure, and these have largely been targeted using small panels of proteins or smaller, isolated sample sets. A statistically robust association has been described between metformin exposure and serum Growth differentiation factor 15 (GDF15) concentrations, first discovered using a Luminex panel of 237 proteins in the ORIGIN cohort.^4^ This observation was extended through mechanistic rodent studies establishing that metformin-associated increase in GDF15 resulted in a reduction in food intake and body weight.^5^ More recently, Gummesson et al. carried out a more comprehensive proteomic analysis following metformin treatment. This further showed that GDF15 was increased following metformin treatment in addition to identifying other proteins significantly altered by metformin exposure; for example, EpCAM was reduced in those treated with metformin.^6^

To better understand how metformin alters the circulating proteome, here we extend prior analyses in a comparatively large sample of 2057 participants from different cross-sectional and from longitudinal studies of metformin exposure, using two commonly used proteomic panels.

## Methods

**Fig. 1:**
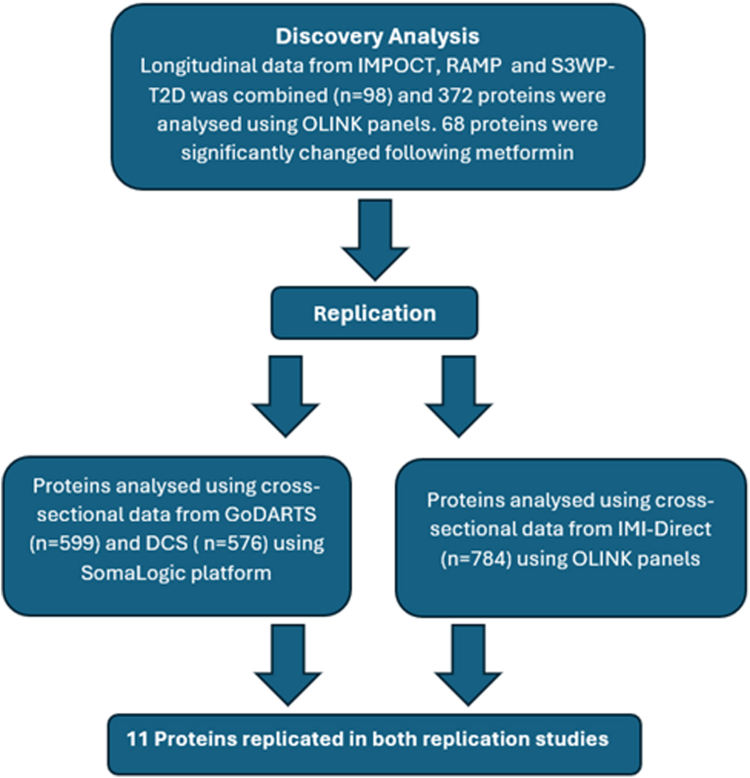
Flowchart showing the study design which identifies many proteins that are significantly changed following exposure to metformin.

We undertook initial analysis in a combined longitudinal dataset that includes the previously reported S3WP-T2D cohort^6^ but expanded with two additional cohorts (IMPOCT and RAMP) to increase statistical power. Replication used cross-sectional data from the IMI-RHAPSODY study (including two cohorts GoDARTS and DCS) and the IMI-DIRECT study. Design of the study is shown in Fig. 1.

### Ethics

The studies described were approved by the local medical ethics committees (Supplementary Table S1). All participants provided written informed consent before the start of the studies. The research conformed to the ethical principles for medical research involving human participants outlined in the declaration of Helsinki.

### Discovery cohorts

Individual level longitudinal data were combined from the following 3 cohorts.

### S3WP-T2D

This study was carried out to elucidate the changes in the proteome in the early stages of diabetes and how the proteome is affected by diabetes treatments including metformin.^6^ 52 previously undiagnosed individuals were identified as having type 2 diabetes through a screening programme, and as a result were recruited for the study. Individuals were excluded if they had a pre-existing disease which would affect their ability to participate, severe hyperglycaemia needing hospitalisation or immediate insulin therapy, or a major surgical procedure or trauma within the prior 4-weeks. Enrolled participants were treated for diabetes via first-line therapy; weight management and exercise with or without metformin, which was decided by a doctor. Protein levels in the blood were measured at baseline, one month and three months using Olink (details see proteomics section). Of the 52 participants, 51 completed the 3-month follow-up visit, and for 3 participants’ plasma samples were not available for the 1-month visit. This left data for 48 participants for statistical analysis.

### IMPOCT

The IMPOCT study was designed to investigate the impact of the *SLC22A1* (*OCT1*) genotype and OCT1 inhibiting drugs on an individual's ability to tolerate metformin. For this analysis, only data from when individuals were treated with metformin or placebo were utilised, and not data from individuals on OCT1 inhibiting drugs. 38 healthy participants without diabetes were recruited for this study. They were on metformin for 4 weeks, titrated to a maximum dose of 1000 mg BD which they took for the final week of the study. Protein levels in the blood were measured at baseline and after the 4 weeks of metformin treatment using various Olink panels (details see proteomics section).

### RAMP

The RAMP study^7^ was designed to investigate the response of individuals with ataxia telangiectasia to metformin and pioglitazone. For this analysis, we only utilised data from control participants (without ataxia telangiectasia) on metformin (not pioglitazone). Metformin therapy was initiated in 12 non-diabetic, healthy controls, who had not previously been treated with the drug. Metformin administration last 8-weeks, titrated to a maximum dose of 1000 mg BD for the final 4 weeks of the study. Protein levels in the blood were measured at baseline and after the 8 weeks of metformin treatment using various Olink panels (details see proteomics section).

### Replication in cross sectional studies

#### Olink replication

This replication uses data from the IMI-DIRECT (DIabetes REsearCh on patient straTification) study: This cohort included 784 patients with recently diagnosed type 2 diabetes. The mean age at inclusion was 62 years with the youngest 35 years old at baseline. Participants were diagnosed 0–24 months before recruitment, were on lifestyle and/or metformin treatment only, and had glycated haemoglobin (HbA_1c_) < 60.0 mmol/mol (<7.6%) within the previous three months.^8^ Protein levels were measured using various Olink panels (details see proteomics section).

#### SomaLogic replication

This study consists of two cohort studies, GoDARTS and DCS together as part of the IMI-RHAPSODY study.^9^

#### GoDARTS

The Genetics of Diabetes Audit and Research Tayside Study (GoDARTS) is a cohort of ∼8000 individuals with T2D.^10^ Laboratory measurements were non-fasted. For SomaLogic analysis, samples from 599 patients were selected age >35 years, GAD antibody negative, with blood sampled close to diagnosis (median diabetes duration 1.4 years).

#### DCS

The Hoorn Diabetes Care System (DCS) cohort is a prospective cohort with currently over 14,000 individuals with routine care data. In 2008–2014, additional blood sampling was done in 5500 participants, who provided written informed consent. These samples were used for this study. For SomaLogic analysis, samples from 576 patients were selected age >35 years, GAD antibody negative, with blood sampled close to diagnosis (median diabetes duration 2.6 years).^11^

Protein levels in both cohorts were measured simultaneously using a SomaLogic panel (details see proteomics section).

### Proteomics assays

We used two complementary affinity proteomics approaches to determine the relative levels of circulating proteins in blood samples, Olink and SomaLogic respectively.^12^

For the discovery analysis, after proteins were removed following quality control and to ensure each protein was available in all three cohorts, 372 proteins were analysed in the combined analysis of S3WP-T2D, IMPOCT and RAMP. These originated from the five Olink Target96 panels common across the 3 cohorts (Cardiometabolic, Cardiovascular II, Cardiovascular III, Development and Metabolism).

For replication analyses, the same 372 proteins were available in Olink replication set cohort using the same 5 Olink Target96 panels. For the SomaLogic replication set, the SomaLogic platform was used to measure 1195 proteins (SomaLogic version 1.3). 159 proteins overlapped with the 372 discovery Olink proteins.

### Statistics

This study was based on reusing already existing data and as such there were no special inclusion or exclusion criteria applied specifically for this study nor an a priori power calculation to determine the optimal sample size.

### Discovery: longitudinal Olink analysis

The longitudinal data from the S3WP-T2D cohort described by Gummesson et al.^6^ were combined with the longitudinal data from IMPOCT and RAMP. In these cohorts, Olink panels were used to measure proteins before and after metformin exposure.

Statistical analysis was performed using R Studio version 4.1.2. Proteins were analysed using linear mixed models, with the R package *LmerTest*. Within these models, metformin dose, age, sex and study name were included as fixed-effects, and the participant was included as a random-effect. In a sensitivity analysis the linear mixed model was also run using study name as a random effect and similar results were observed (Correlation between the Beta's of both models was r^2^ = 0.999). Metformin doses are shown in Supplementary Table S2. Dosing in S3WP-T2D was variable due to patients being treated as per guidelines. For the linear mixed model, dosing was collapsed to 0 if they were not on metformin, 1 if they were on a lower dose (500 mg or 1000 mg daily) and 2 if they were on a higher dose (1500 mg or 2000 mg daily), to account for variability in metformin dosage and treatment. P-values were adjusted using the Bonferroni method for multiple test correction, and those that remained statistically significant were taken forward for replication in the two cross-sectional studies. We repeated the analysis to show the S3WP-T2D cohort analysed against the combined dataset of IMPOCT and RAMP to highlight the results in people with diabetes versus controls.

### Sex stratified analyses, and adjustment for BMI and HbA1c change

The analyses for the discovery cohorts were also repeated stratified by both sexes as obtained from the study records. We subsequently analysed sex stratified results for the 11 proteins robustly replicated across all replication cohorts.

To investigate how the change in protein levels with metformin change could have been consequent upon or associated with HbA1c or BMI changes seen with metformin exposure we adjusted for BMI change and HbA1c where possible. BMI after metformin treatment was not available in IMPOCT therefore BMI adjustment was studied in a combined dataset of S3WP-T2D and RAMP. Analysis was again completed using a linear mixed model with BMI, metformin dose and study name as fixed effects and the study individual as a random effect. HbA1C data was only available before and after metformin treatment in S3WP-T2D. The 68 significantly changed proteins in the combined analysis were analysed in S3WP-T2D with and without HbA1C adjustment. We again used metformin dose as a fixed effect and study individual as a random effect in a linear mixed model, and added HbA1C as a fixed effect when adjusting for HbA1C.

### Gene-set enrichment analysis for proteins associated with metformin exposure

We used the *enrichR* package in R to evaluate which tissues were enriched, based upon the change in proteins in response to metformin treatment in the longitudinal studies. Only proteins were included with an adjusted P-value smaller than 0.05/372. Proteins were converted to gene symbols and upregulated and downregulated proteins were tested separately. For the enrichment, P-values were FDR adjusted and a P_FDR_ <0.05 was considered significant.

### Replication: cross sectional Olink and SomaLogic analysis

#### Replication using Olink

A linear mixed model with metformin exposure (Y/N) was applied using the *lmer* function of the R package lme4. In this model, the Olink NPX data was adjusted by information related to the donor (age at sampling, sex), the sampling event (date, centre (random effect) as well as technical aspects (assay plate).

#### Replication using SomaLogic

We undertook linear regression using the biomarker as the dependent variable, with metformin exposure (Y/N) as an independent variable, adjusted for age and sex. This was done for both DCS and GoDARTS and then data were combined using random effects meta-analysis.

Metformin: Protein associations were considered to replicate if they were directionally consistent and with a p < 0.05 in both replication data sets.

### Sex stratified analyses

The above described analyses were repeated stratified by sex for the discovery cohorts and for the subsequent 11 robustly replicated proteins.

### Role of the funders

The funders of this study had no role in the design, data collection, data analysis, data interpretation, or writing of this manuscript.

## Results

### Discovery: longitudinal metformin exposure

**Fig. 2:**
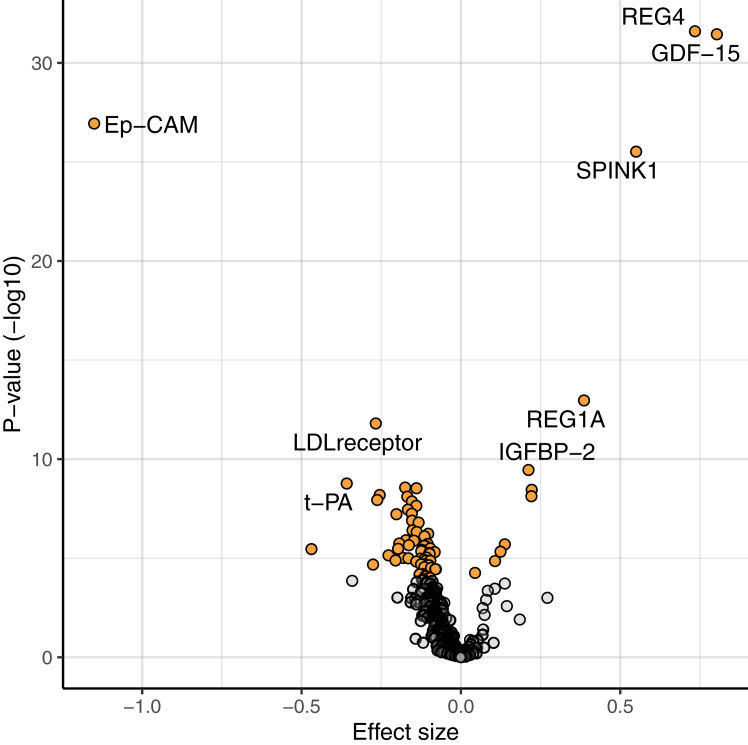
**Effect of metformin on plasma protein levels in the discovery analysis**. Volcano plot showing if protein concentrations are significantly increased or decreased following metformin treatment in the longitudinal Olink analysis (n = 98). Estimate (beta coefficient) is plotted on the x axis and -log10 of the unadjusted p value (calculated from the linear mixed-model) is plotted on the y axis. Proteins with an adjusted p-value (Bonferroni method) of less than 0.05 are represented by a yellow dot and all other non-significantly changed proteins are represented by a grey dot. Proteins which have an increased concentration following metformin treatment have a positive effect size whereas proteins which have a decreased concentration following metformin treatment have a negative effect size.

**Fig. 3:**
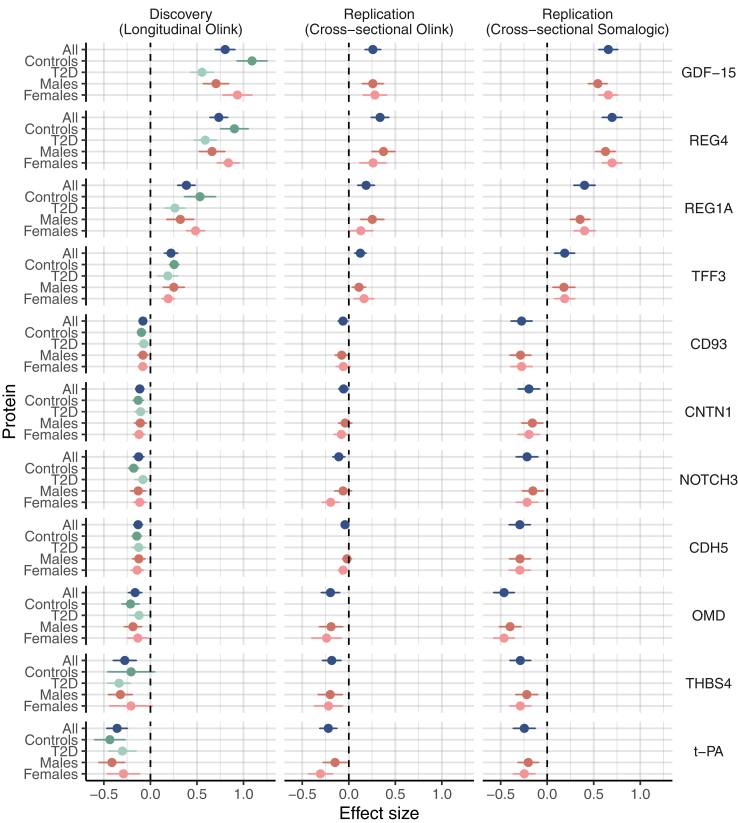
Comparison of effect size across the three studies showing the 11 protein-metformin associations that were replicated in both replication studies (all P ≤ 0.05). Data represent mean ± SE. X-axis, effect size; y-axis, protein. In dark blue the main result for each cohort is shown. Discovery (n = 98), Replication Olink (n = 784) and replication SomaLogic (n = 1175). Green colours represent the results of analysis stratified by diabetes status (dark green = controls, n = 50; light green = T2D, n = 48). Results for the replication cohorts aren't shown as these consist of persons with T2D only. Results of sex stratified analyses where shown as dark red = men and lighter red = women. Discovery (men, n = 52; women, n = 46), Replication Olink (men, n = 449; women, n = 335) and replication SomaLogic (men, n = 679; women, n = 496).

We analysed protein concentrations longitudinally in individuals before metformin treatment and after metformin initiation in 3 clinical trials (Supplementary Data S1). Baseline characteristics of included cohorts are shown in Supplementary Table S2. After Bonferroni correction, 68 proteins (18% of measured proteins on the Olink platform) were found to be significantly associated with metformin treatment (Supplementary Table S3) and are represented in the volcano plot (Fig. 2). The top 8 most significant proteins are labelled in the figure (smallest adjusted p value) and are as follows from most statistically significant to least significant: REG4, GDF-15, EpCAM, SPINK1, REG1A, LDL receptor, IGFBP-2 and t-PA. When analysing this data in S3WP-T2D (all people with diabetes) versus a combined analysis of IMPOCT and RAMP (people without diabetes), the direction of effects are very similar (Supplementary Data S2, top_68 r^2^ = 0.81 and Fig. 3). However, as shown in Fig. 3 it seems the effect sizes are in general slightly stronger in the controls.

Analysis of the longitudinal data including adjustment for BMI change and HbA1c are shown in Supplementary Tables S4 and S5. Because of the reduction in sample size (IMPOCT could not be included as no BMI on treatment was recorded) only 30 proteins were associated with metformin exposure after Bonferroni adjustment. Adjusting for BMI change after metformin initiation had limited impact on these associations with only four out of 68 signals with a beta differing by >25%—i.e. none of our 11 top protein-metformin associations showed major differences after including BMI change in the model (Supplementary Table S4). Considering HbA1c change, which was only available in the S3WP-T2D discovery cohort (n = 48), 19 signals showed a beta difference >25% with or without HbA1c change adjustment (Supplementary Table S5). Adjusting for HbA1c change had little impact on our 11 top signals (all <15%) except for Notch3 for which the association with metformin exposure was markedly increased (+53%, Supplementary Table S5).

In a gene-set enrichment analysis (Supplementary Table S6), upregulated proteins were enriched for colon (OR = 20.4, Padj = 6.49 × 10^−5)^, based on overlap with REG4, REG1A, GDF15, TFF3, SPINK1, CCL15, PIGR and Gal-4. Downregulated proteins were enriched for omentum (OR [95% CI] = 5.2 [2.9–9.1], Padj = 2.93 × 10^−6^) and liver (OR [95% CI] = 4.84 [2.7–8.5], Padj = 7.52 × 10^−6^).

### Replication: cross sectional metformin exposure

Replication was undertaken in two cross sectional replication sets using either of the two proteomics platforms. Baseline characteristics of the study participants are shown in Supplementary Table S2.

From the discovery, all 68 significant longitudinal protein-metformin associations were taken forward for replication analyses, as shown in Supplementary Table S3 and Supplementary Data S3 and S4 for both platforms. Among these were 11 protein-metformin associations replicated in both replication studies (REG4, GDF15, REG1A, t-PA, TFF3, CDH5, CNTN1, OMD, NOTCH3, THBS4, CD93) and these are shown in Fig. 3. There were 12 proteins that were replicated in the Olink replication but were not measured on the SomaLogic platform (version 1.3), namely EPCAM, SPINK1, SAA-4, COMP, ITGB2, ADGRG2, FAM3C, MERTK, COL1A1, HAOX1, VCAN, TIMD4. Adjusting for BMI and HbA1c in the diabetic replication cohorts made no substantial difference to these results (Correlation between the Beta's of model 1 and model 2: Olink replication r^2^ = 0.99; SomaLogic replication r^2^ = 0.98, Supplementary Data S5 and S6). In the SomaLogic replication cohorts a limited number of participants also used sulfonylurea derivatives and or other diabetes drugs (Supplementary Table S2). In a sensitivity analysis additional adjustment for these potential confounders did not influence the results for the 11 replicated proteins (r^2^ = 1 for the correlation of the beta's with or without adjustment, Supplementary data S7).

### Sex stratified analysis

The sex stratified results are provided in Supplementary Table S7 for the longitudinal discovery cohorts. Results were generally directionally consistent between men and women. Focussing on the 11 robustly replicated proteins it appears that GDF-15 and CNTN1 show a consistent, stronger association in women (Fig. 3 and Supplementary Tables S7 and S8).

## Discussion

We have undertaken a comprehensive proteomic analysis of metformin exposure in people with and without diabetes. The concentration of 11 proteins (REG4, GDF15, REG1A, t-PA, TFF3, CDH5, CNTN1, OMD, NOTCH3, THBS4, CD93) were robustly associated with metformin exposure irrespective of the platforms and studies, with a further 12 replicated using the discovery Olink platform (EpCAM, SPINK1, SAA-4, COMP, ITGB2, ADGRG2, FAM3C, MERTK, HAOX1, COL1A1, VCAN, TIMD4). Enrichment analysis showed that the strongest protein-set is of intestinal origin, consistent with the very high concentrations of metformin seen in intestinal epithelial cells.^3^^,^^13^

In the recent paper reporting large-scale proteomic analysis in UK Biobank and an Icelandic cohort, a cross-platform comparison was made across Olink and SomaLogic platforms.^12^ Each protein was given a confidence tier –where tier 1 had a cis-pQTL on two platforms with strong correlation; tier 2 had a cis-pQTL on one platform only or on two platforms but with weak correlation; tier 3 did not have a cis-pQTL on either the Olink or SomaLogic platform. In this context it is interesting to note that our 11 robustly replicated proteins were from tier 1. The three proteins (Gal-4, TF, NOV) that were associated with metformin exposure in both the discovery and replication studies using Olink that were not replicated using the SomaLogic panel and therefore did not pass our replication criteria were in tier 2 (Supplementary Table S3). Our data support that findings associated with the tier 2 proteins should be interpreted with caution and highlights the benefit of cross-platform replication.

In an earlier study by Gummesson et al., metformin was reported to be associated with increased REG4, CPA2, GDF15 and a reduction in EpCAM and PCDH17.^6^ Here, we included the same data alongside two additional longitudinal discovery cohorts. This greatly increased the reliability of our findings. We reproduced the REG4, GDF15 and EpCAM associations in our discovery analysis and replicated these in cross-sectional analyses. Our data also confirms the already robust literature that metformin increases serum GDF-15. GDF-15 is a protein that increases in concentration owing to cellular stress caused by mitochondrial dysfunction, hypoxia, and exercise.^14^ It has been previously shown that the intestine (particularly the lower small intestine and colon) was a main site of increased GDF-15 expression following metformin treatment.^5^ However interestingly a study that used a tissue specific silencing approach showed that up-regulation of renal GDF-15 by metformin (given intravenously) may mediate some of metformin's effects on feeding and weight.^15^ The strong association of intestinal-related proteins with metformin treatment may simply reflect the high exposure of intestinal epithelial cells to metformin and does not necessarily implicate these proteins as mediating any beneficial or potentially harmful effects of metformin. Where data were available, we have investigated whether the association of metformin exposure with protein levels was attenuated when adjusting for HbA1c change or BMI change, as this would support either that the metformin associated protein change is mediating metformin action, or that the protein change is an indirect effect of metformin mediated via its effects on weight or HbA1c. A good example of this is for leptin: leptin concentrations are strongly negatively associated with metformin exposure, yet when adjusting for change in BMI with metformin treatment this association is markedly attenuated suggesting that the association of metformin with lower leptin levels is likely mediated via metformin's effect on body weight. It is interesting to note that very few of the top 11 metformin-protein associations were attenuated by adjusting for either BMI change or HbA1c change which could be consistent with these reflecting intestinal exposure and not mediating metformin's effects. Results in people with or without diabetes are in general directionally consistent although the metformin-protein association seems greater in the controls than those without diabetes—this may reflect the fact that we are comparing a short term acute ‘clean’ intervention in those without diabetes than the longer-term intervention in those with diabetes. As well as the fact that metformin's pharmacological role differs in people with and without diabetes. However, other study designs are needed to investigate this further.

Whilst we cannot conclude that the intestinal signature for the metformin proteome mediates metformin action, it is important to be aware of these strong associations as they may be important confounders in any proteomic analysis in cohorts of metformin-treated individuals, and in clinical settings where protein concentrations are used as biomarkers of treatment response or disease progression, such as a tumour marker. For example, REG4 is a postulated tumour marker for pancreatic adenocarcinoma,^16^ gastric and colorectal cancer,^17^ EpCAM is a well-known tumour marker associated with many cancers including colorectal, ovarian and breast cancers^18^ and REG1A has been recently associated with the development of pancreatic cancer.^19^

The use of two proteomic platforms in both cross-sectional and interventional studies, totalling 2057 participants is a major strength of our study. However, we recognise there are limitations. Firstly, newer proteomic panels include substantially more proteins (e.g. Olink Explore HT measures >5300 proteins, and SomaScan measures 11,000 proteins). Secondly whilst we combine 3 studies that measure proteins before and after metformin initiation, the number of participants in these longitudinal studies remains small. Thirdly, our study design prohibits us to infer differences in biological processes of metformin in those with and without diabetes. Fourthly, given the different study designs, it was not possible to consider a meta-analysis across all the studies. Instead, we used the most powered design (longitudinal repeated measures) as our discovery, limiting the cross-sectional cohorts to replication. We are aware that lack of replication could simply reflect lack of power or the difference in cohort and study design, but where we have replication across multiple cohorts and multiple proteomic platforms, we are confident that these results are robust. Fifthly, our results are based on the analysis of data from participants with a European white background and as such might not be representative for persons with other ethnic backgrounds. Finally, whilst we establish many robust signals, we do not describe causal mechanisms for these associations. This will require further work with, for example, mouse models as has been demonstrated for the mechanistic contribution of GDF15 to metformin action.^5^

In conclusion, we have carried out a comprehensive and systematic study on changes to the circulating proteins following metformin treatment. We show that the proteomic signature of metformin highlights the potential for metformin-induced bias in human proteomic studies, indicating the need to adjust for metformin treatment in future studies.

## Contributors

BC, LM, RCS, JMS, AG, LMtH, ERP designed the study and wrote the manuscript. BC, KFB, LD, JAK, RCS, LMtH analysed the data. M-GH, RWK, GB, JWJB, PME, PWF and all co-authors critically reviewed and approved the final manuscript. The IMI-DIRECT and IMI-RHAPSODY consortia contributed by providing access to their proteomic and metformin data. LMtH and ERP have accessed and verified the underlying data and are the guarantors of this work and, as such, take responsibility for the integrity of the data and the accuracy of the data analysis.

## Data sharing statement

All summary results for the discovery and replication studies are provided in the Supplementary Data Files. The generated individual level proteomic data and linked clinical data in S3WP-T2D, IMPOCT, RAMP, IMI-RHAPSODY (DCS, GoDARTS; EGAD00010002447) and IMI-DIRECT are considered sensitive patient data and cannot be made publicly available in compliance with the European privacy regulations governed by GDPR and according to limitations included in the informed consents signed by the study participants. Data can be available by request to the corresponding authors (E.Z.Pearson@dundee.ac.uk or lmthart@lumc.nl). The IMI-DIRECT data access policy is available at https://directdiabetes.org. Requests will be evaluated by the local data owners for compliance with the informed consents and only approved after signing a data access agreement. Requests should include name and contact details of the person requesting the data, which data and clinical variables are requested and the purpose of requesting the data.

## Declaration of interests

JS received support for attending meetings and/or travel from Luminex and Olink. RWK is currently an employee of Novo Nordisk A/S and owns stock of Novo Nordisk A/S. There are no other relevant conflicts of interest to disclose for this study.

## Appendix A Supplementary data

Supplementary Material

IMI-DIRECT Group Author List

IMI-RHAPSODY Group Author List
