# Supplementary material for: The influence of metformin treatment on the circulating proteome
Source: eBioMedicine. 2025 Jul 19;118:105859. doi: 10.1016/j.ebiom.2025.105859 (PMC12301841; doi:10.1016/j.ebiom.2025.105859)
Supplement: Supplementary Material [file mmc2.docx]

**Supplementary figures and tables**

S Table 1 – Ethics approvals

S Table 2 – Characteristics of cohort participants.

S Table 3 - Significantly changed proteins following metformin exposure in each of the 3 analyses.

S Table 4 - Analysis of the 68 significantly changed proteins in Discovery analysis in S3WP-T2D and RAMP following adjustment for BMI

S Table 5 - Analysis of the 68 significantly changed proteins in Discovery analysis in S3WP-T2D following adjustment for HbA1C

S Table 6 - Gene set enrichment analysis by tissue for proteins significantly up and down regulated by metformin exposure.

S Table 7 - Comparison of Longitudinal Olink analysis between men and women.

S Table 8 – Sex stratified analysis in the Olink and Somalogic replication cohorts

**S Table 1. Ethics approvals**

| **Study Name** | **Name of the ethics board** | **Registration number** |
| --- | --- | --- |
| **Olink Discovery** |  |  |
| S3WP-T2D | Ethical Review Board of Göteborg, Sweden | 448-16 |
| IMPOCT | East of Scotland Ethics Committee | 15/ES/0189 |
| RAMP | East of Scotland Ethics Committee | 16/ES/0056 |
| **Olink replication IMI-DIRECT** |  |  |
| ULUND | Regional ethics testing board in Lund | 2012/155; 2012/779; 2015/843 |
| UCPH | The Scientific Ethics Committee E for the Capital Region of Denmark | H-1-2012-100 |
| VUmc | Medical ethics committee Amsterdam University Medical Center | 2012/222 |
| UK-cohorts (Newcastle, Exeter, Dundee) | NRES Research Ethics Committee North East | 2011DIRECT02; 12/NE/0132 |
| **Somalogic replication IMI_Rhapsody** |  |  |
| DCS | Medical ethics committee Amsterdam University Medical Center | 2007/57 |
| GoDARTS | Tayside medical ethics committee | 053/04 |

S Table 2. Characteristics of cohort participants.

|  | **Discovery: Longitudinal Olink** | | | **Replication: Cross sectional Somalogic** | | **Replication: Cross sectional Olink** |
| --- | --- | --- | --- | --- | --- | --- |
| **Clinical Variable** | **S3WP-T2D** | **IMPOCT** | **RAMP** | **GoDarts** | **DCS** | **IMI-DIRECT** |
| Number of Participants, n | 48 | 38 | 12 | 599 | 576 | 784 |
| Type 2 Diabetes Present, Y/N | Y | N | N | Y | Y | Y |
| Number treated in each study | 48 | 38 | 12 | 266(44.4) | 376 (65.3) | 273 (34.8) |
| Etnicity | European white | European white | European white | European white | European white | European white |
| Time between metformin initiation and on-treatment samples | 1 and 3 months | 4 weeks | 8 weeks | Cross sectional | Cross Sectional | Cross Sectional |
| Dose of metformin (mg) | 2000 (1000-2000) | Titrated to 1000mg BD for final week | Titrated to 1000mg BD taken for final 4 weeks | 1000 (1000-1500) | 1000 (500-1750) | 1000 (500-1000) |
| Men, n (%) | 27 (60.4) | 19 (50) | 6 (50) | 354 (59.1) | 325 (56.4) | 449 (57.3) |
| Age, years  Men  Women | 58.9 (4.6)  58.5 (4.9)  59.5 (4.0) | 60.0 (7.3)  59.8 (7.9)  60.2 (6.6) | 22.3 (2.7)  21.1 (1.2)  23.3 (3.2) | 61.4 (11.0)  61.2 (10.8)  61.5 (11.4) | 63.2 (10.6)  62.7 (10.4)  63.8 (10.5) | 61.8 (8.1)  61.7 (7.9)  61.9 (8.4) |
| Body Mass Index, kg/m^2^  Men  Women | 31.1 (6.0)  31.5 (5.7)  30.5 (6.3) | 26.9 (3.2)  27.0 (3.5)  26.9 (2.9) | 23.1 (1.9)  23.1 (1.8)  23.1 (2.0) | 32.3 (6.8)  31.5 (5.9)  33.6 (7.7) | 30.3 (5.3)  29.7 (4.8)  31.0 (5.7) | 30.6 (5.1)  30.1 (4.4)  31.2 (5.8) |
| HbA1c (mmol/mol)  Men  Women | 44.4 (8.2)  42.7 (6.5)  46.9 (9.7) | N/A | N/A | 67.2 (22.4)  67.7 (22.5)  66.4 (22.4) | 46.8 (8.6)  46.6 (8.8)  47.2 (8.4) | 46.6 (5.7)  46.2 (6.0)  47.0 (5.5) |
| Change in HbA1c | -5.3 | N/A | N/A | na | na | na |
| Change in BMI | -1.1 | N/A | -0.28 | na | na | na |
| DM treatment  - Diet  - Metformin only  - Metformin + SU  - Metformin + other oral  - Other DM drugs | 7  41  0  0  0 | 0  38  0  0  0 | 0  12  0  0  0 | 274  205  32  13  45 | 169  299  68  7  33 | 511  273  0  0  0 |

Characteristics of cohort participants. Values are mean (+-SD) unless otherwise stated.

S Table 3. Significantly changed proteins following metformin exposure in each of the 3 analyses

| **Protein** | **Confidence Tier** | **Longitudinal OLINK BETA** | **Longitudinal OLINK P VALUE** | **Cross-sectional OLINK BETA** | **Cross-sectional OLINK P VALUE** | **Cross-sectional SomaLogic BETA** | **Cross-sectional SomaLogic P VALUE** |
| --- | --- | --- | --- | --- | --- | --- | --- |
| **REG4** | **1** | **0.73** | **2.54E-32** | **0,33** | **1,75E-11** | **0.70** | **6.74E-17** |
| **GDF-15** | **1** | **0.80** | **3.60E-32** | **0,26** | **1,60E-09** | **0.66** | **2.38E-11** |
| **EP-CAM** | **3** | **-1.15** | **1.15E-27** | **-1,04** | **2,40E-40** |  |  |
| **SPINK1** | **2** | **0.55** | **3.02E-26** | **0,40** | **1,32E-20** |  |  |
| **REG1A** | **1** | **0.39** | **1.09E-13** | **0,19** | **4,55E-05** | **0.40** | **7.18E-6** |
| LDLR | 3 | -0.27 | 1.58E-12 | 0,046 | 3,43E-01 | -0.048 | 7.28E-1 |
| IGFBP-2 | 1 | 0.21 | 3.52E-10 | -0,041 | 4,12E-01 |  |  |
| **t-PA** | **1** | **-0.36** | **1.70E-9** | **-0,22** | **3,15E-06** | **-0.25** | **4,46E-5** |
| CDH2 | 3 | -0.17 | 2.72E-9 | 0,081 | 5,85E-02 | -0.088 | 1.50E-1 |
| SEMA7A | 2 | -0.14 | 2.95E-9 | -0,025 | 3,88E-01 |  |  |
| Gal-4 | 2 | 0.22 | 3.52E-9 | 0,24 | 1,86E-07 | 0.072 | 2.37E-1 |
| **SAA4** | **2** | **-0.25** | **6.46E-9** | **-0,13** | **8,06E-03** |  |  |
| **TFF3** | **1** | **0.22** | **7.44E-9** | **0,12** | **5,67E-05** | **0.19** | **5.74E-4** |
| OPN | 1 | -0.17 | 7.86E-9 | -0,081 | 6,58E-02 | -0.06 | 3,36E-1 |
| LEP | 1 | -0.26 | 1.15E-8 | 0,013 | 8,49E-01 | 0.042 | 6.60E-1 |
| FCN2 | 1 | -0.15 | 1.36E-8 | -0,026 | 5,74E-01 | 0.16 | 5.25E-3 |
| ADGRE2 | 1 | -0.14 | 2.28E-8 | -0,005 | 8,82E-01 |  |  |
| DLK-1 | 1 | -0.17 | 3.53E-8 | -0,077 | 1,38E-01 |  |  |
| IGF2R | 1 | -0.15 | 5.70E-8 | 0,021 | 4,51E-01 | -0.13 | 2.84E-2 |
| **COMP** | **1** | **-0.20** | **6.02E-8** | **-0,15** | **2,86E-04** |  |  |
| SELE | 1 | -0.15 | 1.27E-7 | 0,033 | 4,97E-01 | 0.12 | 1.05E-1 |
| **CDH5** | **1** | **-0.13** | **1.59E-7** | **-0,041** | **2,11E-02** | **-0.29** | **2.54E-4** |
| ICAM1 | 2 | -0.15 | 3.93E-7 | 0,006 | 8,57E-01 | 0.066 | 6.50E-1 |
| **ITGB2** | **3** | **-0.14** | **4.76E-7** | -0,10 | 6,66E-04 |  |  |
| TF | 2 | -0.10 | 5.82E-7 | -0,14 | 7,66E-08 | -0.026 | 6.69E-1 |
| **CNTN1** | **1** | **-0.11** | **7.91E-7** | **-0,056** | **3,43E-02** | **-0.20** | **8.07E-4** |
| NOV | 2 | -0.17 | 1.24E-6 | -0,083 | 9,83E-03 | 0.080 | 1.97E-1 |
| **ADGRG2** | **3** | **-0.15** | **1.31E-6** | **-0,084** | **2,87E-04** |  |  |
| FETUB | 1 | -0.19 | 1.81E-6 | -0,017 | 6,38E-01 | -0.12 | 4.05E-2 |
| SIGLEC7 | 2 | -0.10 | 1.92E-6 | 0,047 | 8,87E-02 | -0.015 | 8.05E-1 |
| **FAM3C** | **3** | **0.14** | **1.99E-6** | **0,17** | **3,44E-07** |  |  |
| CD97 | 1 | -0.16 | 2.21E-6 | 0,012 | 7,20E-01 | 0.098 | 1.07E-1 |
| uPA | 2 | -0.11 | 2.39E-6 | -0,041 | 1,90E-01 | -0.15 | 2.42E-1 |
| **MERTK** | **3** | **-0.11** | **2.41E-6** | **-0,061** | **4,71E-02** |  |  |
| TIE1 | 2 | -0.10 | 3.21E-6 | -0,027 | 1,77E-01 | -0.13 | 3.16E-2 |
| CHRDL2 | 1 | -0.20 | 3.34E-6 | -0,087 | 8,15E-02 |  |  |
| **HAOX1** | **3** | **-0.47** | **3.47E-6** | **0,25** | **2,25E-02** |  |  |
| FCGR3B | 2 | -0.12 | 3.76E-6 | -0,067 | 1,26E-01 | -0.035 | 5.59E-1 |
| DPP4 | 2 | -0.12 | 4.45E-6 | -0,043 | 1,46E-01 |  |  |
| PON3 | 3 | 0.12 | 4.67E-6 | -0,079 | 1,03E-01 |  |  |
| AP-N | 3 | -0.08 | 4.91E-6 | -0,043 | 1,13E-01 |  |  |
| LTBR | 3 | -0.10 | 5.48E-6 | -0,030 | 2,64E-01 | 0.11 | 7.37E-2 |
| TYRO3 | 2 | -0.10 | 6.03E-6 | -0,015 | 4,76E-01 | -0.077 | 2.09E-1 |
| SERPINA12 | 1 | -0.23 | 7.14E-6 | -0,14 | 7,65E-02 |  |  |
| **COL1A1** | **1** | **-0.11** | **9.25E-6** | **-0,16** | **3,52E-08** |  |  |
| ENPP7 | 1 | -0.18 | 9.64E-6 | 0,11 | 2,05E-01 | 0.11 | 6.84E-2 |
| **OMD** | **1** | **-0.16** | **1.01E-5** | **-0,20** | **7,87E-05** | **-0.46** | **7.01E-7** |
| **NOTCH3** | **1** | **-0.13** | **1.06E-5** | **-0,11** | **1,03E-03** | **-0.22** | **2.37E-4** |
| **VCAN** | **3** | **-0.12** | **1.20E-5** | **-0,054** | **4,42E-02** |  |  |
| PAI | 1 | -0.21 | 1.30E-5 | 0,073 | 2,42E-01 | -0.21 | 4,61E-3 |
| AOC3 | 3 | -0.11 | 1.33E-5 | -0,010 | 7,20E-01 |  |  |
| BCAM | 2 | -0.10 | 1.35E-5 | 0,005 | 8,56E-01 | -0.13 | 3.17E-1 |
| CCL15 | 1 | 0.11 | 1.39E-5 | 0,038 | 3,44E-01 | 0.15 | 1.05E-2 |
| CD300LG | 3 | -0.14 | 1.47E-5 | -0,049 | 1,50E-01 |  |  |
| SELL | 1 | -0.11 | 2.02E-5 | -0,044 | 1,88E-01 | -0.25 | 2.04E-5 |
| THOP1 | 3 | -0.13 | 2.05E-5 | 0,025 | 5,63E-01 |  |  |
| **THBS4** | **1** | **-0.28** | **2.10E-5** | **-0,18** | **2,74E-04** | **-0.29** | **2.15E-2** |
| CNTN4 | 2 | -0.11 | 2.27E-5 | -0,022 | 4,18E-01 | -0.099 | 9.76E-2 |
| IL18 | 3 | -0.11 | 2.85E-5 | 0,044 | 2,90E-01 |  |  |
| TFPI | 2 | -0.09 | 3.08E-5 | -0,057 | 5,39E-02 | 0.066 | 2.69E-1 |
| ALCAM | 2 | -0.08 | 3.60E-5 | -0,019 | 4,32E-01 | -0.10 | 8.02E-2 |
| **CD93** | **1** | **-0.08** | **3.62E-5** | **-0,062** | **1,75E-02** | **-0.28** | **1.58E-6** |
| PIGR | 2 | 0.04 | 5.51E-5 | 0,019 | 9,67E-02 | -0.097 | 1.66E-1 |
| FUCA1 | 3 | -0.12 | 6.36E-5 | 0,11 | 6,79E-02 |  |  |
| **TIMD4** | **1** | **-0.13** | **6.60E-5** | **-0,11** | **6,84E-03** |  |  |
| CDON | 1 | -0.11 | 8.34E-5 | -0,026 | 2,75E-01 | -0.065 | 2.73E-1 |
| PGF | 2 | -0.10 | 0.0001 | -0,025 | 2,91E-01 | -0.017 | 7.75E-1 |
| CD58 | 2 | -0.12 | 0.00012 | -0,014 | 5,52E-01 |  |  |

Table showing proteins that were significantly changed after metformin in the discovery study, with results presented for both the discovery and two replication studies. The beta and p value of each protein is shown if the protein is included in that study and highlighted red if both replication studies were significant (p<0.05). Proteins highlighted in blue are significant in the one OLINK replication study but the protein was not measured by our Somalogic panel. Confidence tiers are shown based upon the presence of a cis-pQTL across the OLINK and Somalogic platforms as described in this study (https://doi.org/10.1038/s41586-023-06563-x); where tier 1 had cis-PQTL on two platforms with strong correlation; tier 2 had a cis-pQTL on one platform only or on two platforms but with weak correlation; tier 3 did not have a cis-pQTL on either the OLINK or somalogic platform.

Supplementary Table 4. Attenuation of metformin association by adjusting for BMI change

|  | **Unadjusted for weight change** | | | **Adjusted for weight change** | | |  |
| --- | --- | --- | --- | --- | --- | --- | --- |
| **Protein** | **Estimate** | **P value** | **Adj P value** | **Estimate** | **P value** | **Adj P Value** | **Percentage Attenuation** |
| REG4 | 0.64 | 1.97e-20 | 7.34e-18 | 0.62 | 6.00e-20 | 2.23e-17 | -2% |
| GDF-15 | 0.69 | 5.39e-18 | 2.00e-15 | 0.69 | 4.90e-18 | 1.82e-15 | 0% |
| Ep-CAM | -1.25 | 3.41e-22 | 1.27e-19 | -1.25 | 3.25e-22 | 1.21e-19 | 0% |
| SPINK1 | 0.44 | 4.46e-13 | 1.66e-10 | 0.43 | 1.41e-12 | 5.25e-10 | -3% |
| REG1A | 0.33 | 1.41e-09 | 5.24e-07 | 0.32 | 4.03e-09 | 1.50e-06 | -3% |
| LDLR | -0.21 | 6.65e-06 | 0.0025 | -0.18 | 4.16e-05 | 0.015 | -12% |
| IGFBP-2 | 0.21 | 1.15e-06 | 0.00043 | 0.17 | 8.42e-05 | 0.031 | -21% |
| t-PA | -0.33 | 1.37e-06 | 0.00051 | -0.34 | 1.27e-06 | 0.00047 | 1% |
| CDH2 | -0.19 | 1.21e-06 | 0.00045 | -0.17 | 9.41e-06 | 0.0035 | -11% |
| SEMA7A | -0.12 | 1.86e-05 | 0.0069 | -0.11 | 0.00014 | 0.052 | -12% |
| Gal-4 | 0.15 | 0.00073 | 0.27 | 0.15 | 0.00074 | 0.28 | 2% |
| SAA4 | -0.24 | 3.20e-05 | 0.012 | -0.23 | 6.96e-05 | 0.026 | -5% |
| TFF3 | 0.20 | 8.03e-05 | 0.030 | 0.20 | 0.00010 | 0.038 | -1% |
| OPN | -0.18 | 8.07e-07 | 0.00030 | -0.18 | 1.20e-06 | 0.00044 | 0% |
| LEP | -0.19 | 0.00064 | 0.24 | -0.074 | 0.15 | 1 | **-60%** |
| FCN2 | -0.14 | 4.83e-05 | 0.018 | -0.12 | 0.00065 | 0.24 | -15% |
| ADGRE2 | -0.14 | 2.39e-05 | 0.0089 | -0.14 | 6.25e-05 | 0.023 | -5% |
| DLK-1 | -0.17 | 3.56e-05 | 0.013 | -0.16 | 9.98e-05 | 0.037 | -6% |
| IGF2R | -0.17 | 1.40e-06 | 0.00052 | -0.16 | 2.15e-06 | 0.00080 | -3% |
| COMP | -0.19 | 8.07e-05 | 0.030 | -0.18 | 0.00015 | 0.057 | -5% |
| SELE | -0.18 | 7.13e-06 | 0.0027 | -0.13 | 0.00050 | 0.19 | **-26%** |
| CDH5 | -0.14 | 9.85e-05 | 0.037 | -0.13 | 0.00018 | 0.066 | -3% |
| ICAM1 | -0.15 | 0.00021 | 0.079 | -0.14 | 0.00051 | 0.19 | -7% |
| ITGB2 | -0.14 | 1.88e-05 | 0.0070 | -0.13 | 6.03e-05 | 0.022 | -7% |
| TF | -0.12 | 5.46e-06 | 0.0020 | -0.13 | 3.09e-06 | 0.0011 | 4% |
| CNTN1 | -0.11 | 0.00032 | 0.12 | -0.12 | 0.00018 | 0.068 | 6% |
| NOV | -0.15 | 0.00094 | 0.35 | -0.14 | 0.0019 | 0.72 | -7% |
| ADGRG2 | -0.16 | 0.00011 | 0.041 | -0.17 | 4.37e-05 | 0.016 | 7% |
| FETUB | -0.15 | 0.0034 | 1 | -0.14 | 0.0042 | 1 | -2% |
| SIGLEC7 | -0.11 | 9.90e-05 | 0.037 | -0.11 | 0.00031 | 0.12 | -8% |
| FAM3C | 0.14 | 0.00016 | 0.058 | 0.14 | 0.00015 | 0.055 | 1% |
| CD97 | -0.18 | 0.00012 | 0.044 | -0.17 | 0.00023 | 0.087 | -5% |
| uPA | -0.10 | 0.00090 | 0.33 | -0.10 | 0.00087 | 0.32 | 2% |
| MERTK | -0.096 | 0.0026 | 0.97 | -0.098 | 0.0025 | 0.92 | 2% |
| TIE1 | -0.081 | 0.0026 | 0.97 | -0.076 | 0.0048 | 1 | -7% |
| CHRDL2 | -0.19 | 0.00095 | 0.35 | -0.18 | 0.0014 | 0.54 | -3% |
| HAOX1 | -0.50 | 2.99e-05 | 0.011 | -0.45 | 0.00022 | 0.080 | -11% |
| FCGR3B | -0.11 | 0.0013 | 0.48 | -0.10 | 0.0033 | 1 | -7% |
| DPP4 | -0.12 | 0.00095 | 0.35 | -0.12 | 0.0018 | 0.66 | -6% |
| PON3 | 0.14 | 0.00018 | 0.066 | 0.11 | 0.0034 | 1 | -23% |
| AP-N | -0.074 | 0.0021 | 0.78 | -0.073 | 0.0027 | 0.99 | -1% |
| LTBR | -0.10 | 0.00028 | 0.10 | -0.10 | 0.00036 | 0.14 | -1% |
| TYRO3 | -0.10 | 0.00051 | 0.19 | -0.098 | 0.00099 | 0.37 | -5% |
| SERPINA12 | -0.25 | 0.00010 | 0.039 | -0.22 | 0.00074 | 0.27 | -11% |
| COL1A1 | -0.11 | 0.00069 | 0.26 | -0.10 | 0.0011 | 0.41 | -4% |
| ENPP7 | -0.18 | 0.00098 | 0.37 | -0.13 | 0.015 | 1 | **-26%** |
| OMD | -0.13 | 0.0040 | 1 | -0.14 | 0.0031 | 1 | 5% |
| Notch3 | -0.11 | 0.0056 | 1 | -0.12 | 0.0038 | 1 | 6% |
| VCAN | -0.11 | 0.0010 | 0.39 | -0.12 | 0.00047 | 0.17 | 8% |
| PAI | -0.28 | 2.59e-05 | 0.0096 | -0.24 | 0.00016 | 0.060 | -12% |
| AOC3 | -0.097 | 0.0040 | 1 | -0.093 | 0.0061 | 1 | -4% |
| BCAM | -0.097 | 0.00065 | 0.24 | -0.099 | 0.00062 | 0.23 | 2% |
| CCL15 | 0.087 | 0.0062 | 1 | 0.082 | 0.012 | 1 | -6% |
| CD300LG | -0.13 | 0.0019 | 0.69 | -0.13 | 0.0020 | 0.76 | 0% |
| SELL | -0.10 | 0.0052 | 1 | -0.091 | 0.0098 | 1 | -7% |
| THOP1 | -0.12 | 0.0017 | 0.62 | -0.11 | 0.0038 | 1 | -8% |
| THBS4 | -0.29 | 3.89e-06 | 0.0014 | -0.27 | 1.29e-05 | 0.0048 | -9% |
| CNTN4 | -0.088 | 0.0026 | 0.97 | -0.086 | 0.0036 | 1 | -2% |
| IL18 | -0.12 | 0.0012 | 0.44 | -0.11 | 0.0022 | 0.81 | -4% |
| TFPI | -0.13 | 1.87e-05 | 0.0069 | -0.12 | 4.25e-05 | 0.016 | -4% |
| ALCAM | -0.083 | 0.0012 | 0.43 | -0.080 | 0.0019 | 0.72 | -4% |
| CD93 | -0.073 | 0.0054 | 1 | -0.072 | 0.0068 | 1 | -2% |
| PIgR | 0.05 | 0.00018 | 0.069 | 0.054 | 0.00014 | 0.054 | 2% |
| FUCA1 | -0.088 | 0.018 | 1 | -0.014 | 0.71 | 1 | **-84%** |
| TIMD4 | -0.11 | 0.0077 | 1 | -0.099 | 0.020 | 1 | -13% |
| CDON | -0.097 | 0.0085 | 1 | -0.095 | 0.0099 | 1 | -2% |
| PGF | -0.093 | 0.0039 | 1 | -0.092 | 0.0046 | 1 | -2% |
| CD58 | -0.13 | 0.00091 | 0.34 | -0.14 | 0.00085 | 0.32 | 1% |

Supplementary Table 4 showing a combined analysis of RAMP and S3WP-T2D. Results are shown for an unadjusted analysis, and following adjustment for BMI change. Any Bonferroni adjusted P value less than 0.05 is highlighted in red. Percentage attenuation of the estimate following BMI adjustment is also shown; >25% in bold).

S Table 5. Attenuation of metformin association by adjusting for HbA1C

|  | **Unadjusted for HbA1C** | | | **Adjusted for HbA1C** | | |  |
| --- | --- | --- | --- | --- | --- | --- | --- |
| **Protein** | **Estimate** | **P value** | **Adj P value** | **Estimate** | **P value** | **Adj P value** | **Percentage Attenuation** |
| REG4 | 0.35 | 1.81e-17 | 6,73e-15 | 0.33 | 1.85e-14 | 6,88e-12 | -5% |
| GDF-15 | 0.34 | 7.44e-17 | 2,77e-14 | 0.34 | 3.73e-15 | 1,39e-12 | 1% |
| Ep-CAM | -0.77 | 3.17e-21 | 1,18e-18 | -0.75 | 1.36e-20 | 5,06e-18 | -2% |
| SPINK1 | 0.25 | 7.61e-12 | 2,83e-09 | 0.26 | 2.81e-11 | 1.00e-08 | 3% |
| REG1A | 0.16 | 7.31e-07 | 0.00034 | 0.16 | 4.99e-06 | 0.0023 | 1% |
| LDLR | -0.13 | 1.17e-05 | 0.0054 | -0.075 | 0.013 | 1 | **-42%** |
| IGFBP-2 | 0.12 | 3.54e-05 | 0.016 | 0.077 | 0.016 | 1 | **-34%** |
| tPA | -0.2 | 5.68e-06 | 0.0026 | -0.22 | 3.32e-06 | 0.0015 | 8% |
| CDH2 | -0.12 | 2.79e-06 | 0.0013 | -0.099 | 0.00029 | 0.13 | -15% |
| SEMA7A | -0.081 | 1.75e-05 | 0.008 | -0.046 | 0.023 | 1 | **-43%** |
| Gal-4 | 0.085 | 0.0016 | 0.73 | 0.103 | 0.00082 | 0.38 | 22% |
| SAA4 | -0.12 | 0.00077 | 0.36 | -0.086 | 0.017 | 1 | **-27%** |
| TFF3 | 0.12 | 0.00021 | 0.096 | 0.13 | 0.00014 | 0.066 | 5% |
| OPN | -0.073 | 0.0011 | 0.52 | -0.083 | 0.0011 | 0.5 | 14% |
| LEP | -0.11 | 0.00091 | 0.42 | -0.065 | 0.091 | 1 | **-40%** |
| FCN2 | -0.082 | 0.0002 | 0.092 | -0.073 | 0.0046 | 1 | -11% |
| ADGRE2 | -0.08 | 0.00016 | 0.071 | -0.089 | 0.00018 | 0.083 | 12% |
| DLK-1 | -0.092 | 0.00034 | 0.16 | -0.095 | 0.001 | 0.47 | 3% |
| IGF2R | -0.091 | 5.60e-05 | 0.026 | -0.081 | 0.0004 | 0.19 | -10% |
| COMP | -0.11 | 0.00056 | 0.26 | -0.1 | 0.0015 | 0.69 | -3% |
| SELE | -0.11 | 8.53e-06 | 0.0039 | -0.074 | 0.0096 | 1 | **-35%** |
| CDH5 | -0.069 | 0.0017 | 0.78 | -0.075 | 0.0019 | 0.88 | 9% |
| ICAM1 | -0.084 | 0.00052 | 0.24 | -0.078 | 0.0031 | 1 | -7% |
| ITGB2 | -0.11 | 4.18e-07 | 0.00019 | -0.083 | 0.00025 | 0.11 | -22% |
| TF | -0.065 | 0.00021 | 0.097 | -0.073 | 0.00019 | 0.089 | 12% |
| CNTN1 | -0.061 | 0.0016 | 0.72 | -0.071 | 0.00091 | 0.42 | 16% |
| NOV | -0.093 | 0.00072 | 0.33 | -0.082 | 0.0036 | 1 | -11% |
| ADGRG2 | -0.093 | 0.00024 | 0.11 | -0.12 | 1.81e-05 | 0.0083 | **28%** |
| FETUB | -0.08 | 0.0093 | 1 | -0.061 | 0.052 | 1 | -23% |
| SIGLEC7 | -0.07 | 0.00016 | 0.073 | -0.058 | 0.0036 | 1 | -17% |
| FAM3C | 0.081 | 0.00057 | 0.26 | 0.079 | 0.0017 | 0.79 | -2% |
| CD97 | -0.1 | 0.00089 | 0.41 | -0.088 | 0.0084 | 1 | -15% |
| uPA | -0.045 | 0.02 | 1 | -0.053 | 0.017 | 1 | 18% |
| MERTK | -0.041 | 0.036 | 1 | -0.052 | 0.021 | 1 | **25%** |
| TIE1 | -0.045 | 0.009 | 1 | -0.049 | 0.0084 | 1 | 10% |
| CHRDL2 | -0.1 | 0.0043 | 1 | -0.12 | 0.0027 | 1 | 17% |
| HAOX1 | -0.23 | 0.0025 | 1 | -0.16 | 0.073 | 1 | **-33%** |
| FCGR3B | -0.073 | 0.00068 | 0.31 | -0.073 | 0.0034 | 1 | 0% |
| DPP4 | -0.073 | 0.0019 | 0.89 | -0.077 | 0.0029 | 1 | 4% |
| PON3 | 0.079 | 0.001 | 0.47 | 0.03 | 0.26 | 1 | **-62%** |
| AP-N | -0.034 | 0.027 | 1 | -0.044 | 0.012 | 1 | **28%** |
| LTBR | -0.048 | 0.01 | 1 | -0.049 | 0.017 | 1 | 3% |
| TYRO3 | -0.054 | 0.0026 | 1 | -0.056 | 0.0056 | 1 | 3% |
| SERPINA12 | -0.14 | 0.00057 | 0.26 | -0.12 | 0.013 | 1 | -15% |
| COL1A1 | -0.053 | 0.0085 | 1 | -0.041 | 0.054 | 1 | -23% |
| ENPP7 | -0.1 | 0.0033 | 1 | -0.067 | 0.11 | 1 | **-37%** |
| OMD | -0.07 | 0.021 | 1 | -0.066 | 0.056 | 1 | -7% |
| Notch3 | -0.049 | 0.049 | 1 | -0.075 | 0.0072 | 1 | **53%** |
| VCAN | -0.073 | 0.00042 | 0.2 | -0.092 | 5.22e-05 | 0.024 | **26%** |
| PAI | -0.16 | 0.00018 | 0.082 | -0.077 | 0.087 | 1 | **-53%** |
| AOC3 | -0.063 | 0.0035 | 1 | -0.049 | 0.036 | 1 | -22% |
| BCAM | -0.054 | 0.0036 | 1 | -0.034 | 0.091 | 1 | **-37%** |
| CCL15 | 0.039 | 0.043 | 1 | 0.028 | 0.21 | 1 | **-27%** |
| CD300LG | -0.075 | 0.0077 | 1 | -0.06 | 0.048 | 1 | -20% |
| SELL | -0.059 | 0.0061 | 1 | -0.062 | 0.0084 | 1 | 5% |
| THOP1 | -0.08 | 0.0009 | 0.41 | -0.077 | 0.0026 | 1 | -3% |
| THBS4 | -0.2 | 1.40e-07 | 6.43e-05 | -0.17 | 1.52e-05 | 0.007 | -14% |
| CNTN4 | -0.044 | 0.021 | 1 | -0.034 | 0.1 | 1 | -23% |
| IL18 | -0.074 | 0.0016 | 0.73 | -0.07 | 0.0085 | 1 | -5% |
| TFPI | -0.059 | 0.0018 | 0.84 | -0.047 | 0.016 | 1 | -19% |
| ALCAM | -0.049 | 0.0025 | 1 | -0.05 | 0.0057 | 1 | 1% |
| CD93 | -0.04 | 0.014 | 1 | -0.046 | 0.011 | 1 | 15% |
| PIgR | 0.04 | 1.47e-05 | 0.0067 | 0.038 | 7.33e-05 | 0.034 | -5% |
| FUCA1 | -0.051 | 0.03 | 1 | -0.015 | 0.59 | 1 | **-71%** |
| TIMD4 | -0.066 | 0.014 | 1 | -0.03 | 0.32 | 1 | **-55%** |
| CDON | -0.049 | 0.03 | 1 | -0.044 | 0.066 | 1 | -11% |
| PGF | -0.041 | 0.042 | 1 | -0.044 | 0.036 | 1 | 9% |
| CD58 | -0.064 | 0.016 | 1 | -0.06 | 0.029 | 1 | -7% |

Shows the 68 significant proteins in discovery analysed in S3WP-T2D both adjusted and unadjusted for HbA1C. Any Bonferroni adjusted P value less than 0.05 is highlighted in red. Percentage attenuation of the estimate following HbA1C adjustment is also shown.

**S Table 6. Gene set enrichment analysis by tissue for proteins significantly up and down regulated by metformin exposure.**

| **Term** | **Overlap** | **P. Value** | **Adjusted P.value** | **Odds**  **Ratio** | **Direction** |
| --- | --- | --- | --- | --- | --- |
| **OMENTUM** | 23/2316 | 2.71E-8 | 2.93E-6 | 5.21 | Downregulated |
| **LIVER (BULK TISSUE)** | 22/2316 | 1.39E-7 | 7.53E-6 | 4.84 | Downregulated |
| **COLON (BULK TISSUE)** | 8/2316 | 3.82E-6 | 6.49E-5 | 20.4 | Upregulated |
| **GASTRIC EPITHELIAL CELL** | 8/2316 | 3.82E-6 | 6.49E-5 | 20.4 | Upregulated |
| **GASTRIC TISSUE (BULK)** | 8/2316 | 3.82E-6 | 6.49E-5 | 20.4 | Upregulated |
| **SMALL INTESTINE (BULK TISSUE)** | 8/2316 | 3.82E-6 | 6.49E-5 | 20.4 | Upregulated |
| **VALVE** | 19/2316 | 1.23E-5 | 0.00044 | 3.84 | Downregulated |
| **ILEUM (BULK)** | 7/2316 | 5.98E-5 | 0.00068 | 13.4 | Upregulated |
| **LIVER (BULK TISSUE)** | 7/2316 | 5.98E-5 | 0.00068 | 13.4 | Upregulated |
| **LUNG (BULK TISSUE)** | 18/2316 | 4.71E-5 | 0.0013 | 3.54 | Downregulated |
| **PLACENTA (BULK)** | 17/2316 | 0.00017 | 0.0030 | 3.26 | Downregulated |
| **RENAL CORTEX** | 17/2316 | 0.00017 | 0.0030 | 3.26 | Downregulated |
| **COLONIC MUCOSA** | 6/2316 | 0.00066 | 0.0056 | 9.18 | Upregulated |
| **HEPATOCYTE** | 6/2316 | 0.00066 | 0.0056 | 9.18 | Upregulated |
| **ADIPOSE (BULK TISSUE)** | 16/2316 | 0.00055 | 0.0059 | 2.99 | Downregulated |
| **BREAST (BULK TISSUE)** | 16/2316 | 0.00055 | 0.0059 | 2.99 | Downregulated |

Protein identifiers were converted to gene symbols. Enrichment was performed using the enrichR package in R. Upregulated proteins and downregulated proteins were tested separately. An adjusted P-value smaller than 0.05 was considered significant.

Supplementary Table 7 - Comparison of Longitudinal Olink analysis between men and women

| **Men (n=54)** | | | | | **Women (n=44)** | | | |  |
| --- | --- | --- | --- | --- | --- | --- | --- | --- | --- |
| **Protein** | **estimate** | **SE** | **P-value** | **Adj P** | **estimate** | **SE** | **P-value** | **Adj P** | **% difference** |
| REG4 | 0,662 | 0,070 | 4,66E-15 | 1,73E-12 | 0,837 | 0,062 | 1,87E-20 | 6,96E-18 | **26%** |
| GDF-15 | 0,704 | 0,069 | 2,00E-16 | 7,44E-14 | 0,935 | 0,080 | 1,00E-17 | 3,72E-15 | **33%** |
| Ep-CAM | -1,132 | 0,118 | 2,72E-15 | 1,01E-12 | -1,175 | 0,126 | 8,88E-14 | 3,30E-11 | 4% |
| SPINK1 | 0,487 | 0,060 | 3,20E-12 | 1,19E-09 | 0,635 | 0,057 | 1,50E-16 | 5,58E-14 | **30%** |
| REG1A | 0,321 | 0,073 | 3,03E-05 | 1,13E-02 | 0,485 | 0,050 | 3,76E-14 | 1,40E-11 | **51%** |
| LDLR | -0,272 | 0,050 | 4,84E-07 | 1,80E-04 | -0,262 | 0,047 | 5,06E-07 | 1,88E-04 | -4% |
| IGFBP-2 | 0,255 | 0,043 | 6,50E-08 | 2,42E-05 | 0,158 | 0,046 | 9,79E-04 | 3,64E-01 | **-38%** |
| t-PA | -0,414 | 0,071 | 8,74E-08 | 3,25E-05 | -0,290 | 0,089 | 1,79E-03 | 6,66E-01 | **-30%** |
| CDH2 | -0,222 | 0,038 | 9,63E-08 | 3,58E-05 | -0,117 | 0,039 | 3,99E-03 | 1 | **-47%** |
| SEMA7A | -0,139 | 0,032 | 4,42E-05 | 1,64E-02 | -0,139 | 0,029 | 1,19E-05 | 4,43E-03 | 0% |
| Gal-4 | 0,210 | 0,047 | 2,26E-05 | 8,41E-03 | 0,234 | 0,054 | 5,88E-05 | 2,19E-02 | 11% |
| SAA4 | -0,346 | 0,062 | 2,57E-07 | 9,56E-05 | -0,141 | 0,049 | 4,95E-03 | 1 | **-59%** |
| TFF3 | 0,251 | 0,058 | 3,66E-05 | 1,36E-02 | 0,190 | 0,033 | 3,58E-07 | 1,33E-04 | -24% |
| OPN | -0,168 | 0,039 | 3,95E-05 | 1,47E-02 | -0,165 | 0,038 | 5,96E-05 | 2,22E-02 | -2% |
| LEP | -0,283 | 0,058 | 4,84E-06 | 1,80E-03 | -0,233 | 0,066 | 7,94E-04 | 2,95E-01 | -18% |
| FCN2 | -0,205 | 0,039 | 8,87E-07 | 3,30E-04 | -0,095 | 0,030 | 2,18E-03 | 8,12E-01 | **-54%** |
| ADGRE2 | -0,160 | 0,031 | 2,31E-06 | 8,59E-04 | -0,116 | 0,036 | 1,93E-03 | 7,19E-01 | **-28%** |
| DLK-1 | -0,174 | 0,041 | 6,45E-05 | 2,40E-02 | -0,155 | 0,038 | 1,42E-04 | 5,27E-02 | -11% |
| IGF2R | -0,181 | 0,039 | 1,32E-05 | 4,91E-03 | -0,120 | 0,035 | 1,25E-03 | 4,64E-01 | **-34%** |
| COMP | -0,238 | 0,049 | 4,91E-06 | 1,83E-03 | -0,158 | 0,051 | 3,14E-03 | 1 | **-34%** |
| SELE | -0,181 | 0,040 | 1,69E-05 | 6,29E-03 | -0,119 | 0,037 | 2,26E-03 | 8,39E-01 | **-34%** |
| CDH5 | -0,125 | 0,034 | 4,81E-04 | 1,79E-01 | -0,143 | 0,033 | 5,90E-05 | 2,19E-02 | 14% |
| ICAM1 | -0,180 | 0,042 | 4,64E-05 | 1,73E-02 | -0,110 | 0,037 | 3,70E-03 | 1 | **-39%** |
| ITGB2 | -0,153 | 0,038 | 1,08E-04 | 4,00E-02 | -0,121 | 0,036 | 1,34E-03 | 4,98E-01 | -21% |
| TF | -0,133 | 0,028 | 6,75E-06 | 2,51E-03 | -0,065 | 0,027 | 1,94E-02 | 1 | **-51%** |
| CNTN1 | -0,109 | 0,031 | 8,69E-04 | 3,23E-01 | -0,122 | 0,031 | 1,65E-04 | 6,14E-02 | 12% |
| NOV | -0,192 | 0,044 | 3,26E-05 | 1,21E-02 | -0,153 | 0,053 | 5,66E-03 | 1 | -20% |
| ADGRG2 | -0,172 | 0,037 | 1,12E-05 | 4,17E-03 | -0,113 | 0,046 | 1,78E-02 | 1 | **-34%** |
| FETUB | -0,281 | 0,054 | 1,21E-06 | 4,50E-04 | -0,085 | 0,053 | 1,15E-01 | 1 | **-70%** |
| SIGLEC7 | -0,148 | 0,026 | 1,96E-07 | 7,29E-05 | -0,051 | 0,033 | 1,33E-01 | 1 | **-66%** |
| FAM3C | 0,095 | 0,041 | 2,15E-02 | 1 | 0,195 | 0,035 | 6,33E-07 | 2,35E-04 | **105%** |
| CD97 | -0,178 | 0,047 | 2,81E-04 | 1,04E-01 | -0,143 | 0,046 | 2,81E-03 | 1 | -20% |
| uPA | -0,118 | 0,032 | 4,67E-04 | 1,74E-01 | -0,098 | 0,031 | 2,78E-03 | 1 | -17% |
| MERTK | -0,105 | 0,033 | 2,14E-03 | 7,95E-01 | -0,125 | 0,032 | 2,51E-04 | 9,32E-02 | 19% |
| TIE1 | -0,126 | 0,028 | 2,59E-05 | 9,63E-03 | -0,057 | 0,026 | 3,57E-02 | 1 | **-55%** |
| CHRDL2 | -0,242 | 0,046 | 1,14E-06 | 4,24E-04 | -0,149 | 0,071 | 4,05E-02 | 1 | **-38%** |
| HAOX1 | -0,365 | 0,144 | 1,28E-02 | 1 | -0,598 | 0,123 | 8,20E-06 | 3,05E-03 | **64%** |
| FCGR3B | -0,178 | 0,035 | 2,47E-06 | 9,19E-04 | -0,053 | 0,035 | 1,38E-01 | 1 | **-70%** |
| DPP4 | -0,166 | 0,038 | 2,92E-05 | 1,09E-02 | -0,072 | 0,034 | 3,85E-02 | 1 | **-57%** |
| PON3 | 0,122 | 0,035 | 9,00E-04 | 3,35E-01 | 0,126 | 0,039 | 1,97E-03 | 7,32E-01 | 3% |
| AP-N | -0,077 | 0,024 | 2,33E-03 | 8,66E-01 | -0,088 | 0,024 | 6,31E-04 | 2,35E-01 | 14% |
| LTBR | -0,091 | 0,030 | 3,01E-03 | 1,00E+00 | -0,108 | 0,029 | 4,83E-04 | 1,80E-01 | 19% |
| TYRO3 | -0,122 | 0,026 | 9,68E-06 | 3,60E-03 | -0,071 | 0,034 | 4,22E-02 | 1 | **-42%** |
| SERPINA12 | -0,225 | 0,065 | 9,03E-04 | 3,36E-01 | -0,229 | 0,074 | 2,85E-03 | 1 | 2% |
| COL1A1 | -0,126 | 0,029 | 4,80E-05 | 1,79E-02 | -0,086 | 0,038 | 2,93E-02 | 1 | **-32%** |
| ENPP7 | -0,250 | 0,052 | 7,78E-06 | 2,89E-03 | -0,093 | 0,058 | 1,12E-01 | 1 | **-63%** |
| OMD | -0,188 | 0,047 | 1,38E-04 | 5,14E-02 | -0,135 | 0,056 | 1,90E-02 | 1 | **-28%** |
| Notch3 | -0,131 | 0,042 | 2,14E-03 | 7,95E-01 | -0,115 | 0,034 | 1,40E-03 | 5,22E-01 | -12% |
| VCAN | -0,140 | 0,036 | 2,06E-04 | 7,65E-02 | -0,081 | 0,035 | 2,57E-02 | 1 | **-42%** |
| PAI | -0,230 | 0,069 | 1,24E-03 | 4,63E-01 | -0,176 | 0,055 | 2,00E-03 | 7,45E-01 | -23% |
| AOC3 | -0,140 | 0,034 | 6,90E-05 | 2,57E-02 | -0,070 | 0,034 | 4,28E-02 | 1 | **-50%** |
| BCAM | -0,099 | 0,029 | 8,26E-04 | 3,07E-01 | -0,092 | 0,032 | 5,95E-03 | 1 | -7% |
| CCL15 | 0,085 | 0,034 | 1,46E-02 | 1 | 0,136 | 0,032 | 8,06E-05 | 3,00E-02 | **60%** |
| CD300LG | -0,154 | 0,044 | 7,64E-04 | 2,84E-01 | -0,125 | 0,044 | 6,09E-03 | 1 | -19% |
| SELL | -0,139 | 0,036 | 2,05E-04 | 7,63E-02 | -0,079 | 0,036 | 3,10E-02 | 1 | **-43%** |
| THOP1 | -0,162 | 0,039 | 8,44E-05 | 3,14E-02 | -0,080 | 0,041 | 5,24E-02 | 1 | **-51%** |
| THBS4 | -0,323 | 0,065 | 3,94E-06 | 1,47E-03 | -0,209 | 0,117 | 7,86E-02 | 1 | **-35%** |
| CNTN4 | -0,117 | 0,034 | 7,59E-04 | 2,82E-01 | -0,081 | 0,030 | 8,54E-03 | 1 | **-31%** |
| IL18 | -0,125 | 0,036 | 9,72E-04 | 3,61E-01 | -0,102 | 0,039 | 1,04E-02 | 1 | -18% |
| TFPI | -0,117 | 0,029 | 1,06E-04 | 3,95E-02 | -0,066 | 0,033 | 5,11E-02 | 1 | **-44%** |
| ALCAM | -0,084 | 0,026 | 1,94E-03 | 7,22E-01 | -0,070 | 0,025 | 7,42E-03 | 1 | -17% |
| CD93 | -0,081 | 0,028 | 4,65E-03 | 1 | -0,082 | 0,024 | 1,23E-03 | 4,59E-01 | 1% |
| PIgR | 0,050 | 0,015 | 1,42E-03 | 5,27E-01 | 0,037 | 0,015 | 1,44E-02 | 1 | **-26%** |
| FUCA1 | -0,143 | 0,041 | 8,78E-04 | 3,26E-01 | -0,089 | 0,039 | 2,70E-02 | 1 | **-38%** |
| TIMD4 | -0,198 | 0,047 | 6,37E-05 | 2,37E-02 | -0,049 | 0,037 | 1,92E-01 | 1 | **-75%** |
| CDON | -0,117 | 0,038 | 2,77E-03 | 1 | -0,102 | 0,039 | 1,13E-02 | 1 | -13% |
| PGF | -0,117 | 0,034 | 9,56E-04 | 3,56E-01 | -0,069 | 0,033 | 4,05E-02 | 1 | **-41%** |
| CD58 | -0,124 | 0,043 | 4,79E-03 | 1 | -0,110 | 0,041 | 9,15E-03 | 1 | -11% |

Supplementary Table 6 showing the comparison between men and women for the 68 significantly changed proteins in the Longitudinal Olink analysis. Significantly adjusted p values after bonferroni correction (adj p<0.05) are highlighted in bold.

**Supplemental Table 8. Sex stratified analyses in the replication cohorts**

|  | **Cross sectional Olink** | | | |  | **Cross sectional Somalogic** | | | |
| --- | --- | --- | --- | --- | --- | --- | --- | --- | --- |
|  | **Men (n=449)** | | **Women (n=335)** | |  | **Men (n=679)** | | **Women (n=496)** | |
| **Protein** | **Beta** | **P value** | **Beta** | **P value** |  | **Beta** | **P value** | **Beta** | **P value** |
| REG4 | 0,373 | 5,42E-09 | 0,261 | 3,89E-04 |  | 0,627 | 2,70E-16 | 0,787 | 1,74E-22 |
| GDF15 | 0,258 | 9,86E-06 | 0,281 | 1,33E-05 |  | 0,546 | 9,63E-11 | 0,800 | 2,28E-14 |
| REG1A | 0,252 | 6,61E-05 | 0,129 | 0,051 |  | 0,362 | 1,75E-03 | 0,458 | 3,59E-07 |
| t-PA | -0,148 | 0,022 | -0,305 | 5,60E-06 |  | -0,203 | 0,010 | -0,309 | 6,47E-03 |
| TFF3 | 0,109 | 2,21E-03 | 0,164 | 2,36E-03 |  | 0,179 | 4,21E-03 | 0,195 | 0,036 |
| CDH5 | -0,019 | 0,42 | -0,062 | 0,020 |  | -0,293 | 1,93E-04 | -0,307 | 0,026 |
| CNTN1 | -0,039 | 0,27 | -0,08 | 0,041 |  | -0,159 | 0,043 | -0,237 | 7,01E-03 |
| OMD | -0,191 | 2,83E-03 | -0,239 | 3,04E-03 |  | -0,401 | 9,16E-07 | -0,537 | 4,84E-10 |
| NOTCH3 | -0,062 | 0,18 | -0,195 | 3,67E-05 |  | -0,154 | 0,063 | -0,280 | 9,56E-03 |
| THBS4 | -0,200 | 2,52E-03 | -0,218 | 4,71E-03 |  | -0,221 | 0,071 | -0,360 | 2,38E-03 |
| CD93 | -0,077 | 0,030 | -0,059 | 0,13 |  | -0,286 | 2,24E-04 | -0,262 | 2,37E-03 |

Table showing results of the sex stratified analyses in the Olink and Somalogic replication cohorts for the top 11 proteins associated with metformin response.
