# Supplementary material for: The influence of metformin treatment on the circulating proteome
Source: eBioMedicine. 2025 Jul 19;118:105859. doi: 10.1016/j.ebiom.2025.105859 (PMC12301841; doi:10.1016/j.ebiom.2025.105859)
Supplement: IMI-DIRECT Group Author List [file mmc3.docx]

| **First Name** | **Middle initials** | **Surname** |
| --- | --- | --- |
| Moustafa |  | Abdalla |
| Jonathan |  | Adam |
| Jerzy |  | Adamski |
| Kofi |  | Adragni |
| Rosa Lundbye | L. | Allesøe |
| Kristine | H. | Allin |
| Anna | A. | Artati |
| Manimozhiyan |  | Arumugam |
| Naeimeh |  | Atabaki Pasdar |
| Tania |  | Baltauss |
| Karina |  | Banasik |
| Anna |  | Barnett |
| Patrick |  | Baum |
| Jimmy | D. | Bell |
| Susanna |  | Bianzano |
| Roberto |  | Bizzotto |
| Amelie |  | Bonnefond |
| Caroline Anna | A. | Brorsson |
| Andrew | A. | Brown |
| Søren |  | Brunak |
| Louise |  | Cabrelli |
| Robert |  | Caiazzo |
| Henna |  | Cederberg |
| Elizaveta |  | Chabanova |
| Marc |  | Clos-Garcia |
| Matilda |  | Dale |
| David |  | Davtian |
| Adem | Y. | Dawed |
| Federico |  | De Masi |
| Nathalie |  | de Preville |
| Koen | F. | Dekkers |
| Harshal | A. | Deshmukh |
| Christiane |  | Dings |
| Avirup |  | Dutta |
| Beate |  | Ehrhardt |
| Line |  | Engelbrechtsen |
| Rebeca |  | Eriksen |
| Yong |  | Fan |
| Juan |  | Fernandez |
| Jorge |  | Ferrer |
| Hugo |  | Fitipaldi |
| Ian | M. | Forgie |
| Annemette |  | Forman |
| Francesca |  | Frau |
| Philippe |  | Froguel |
| Gary |  | Frost |
| Johann |  | Gassenhuber |
| Giuseppe (Nick) | N | Giordano |
| Toni |  | Giorgino |
| Stephen |  | Gough |
| Harald |  | Grallert |
| Rolf |  | Grempler |
| Lenka |  | Groeneveld |
| Leif |  | Groop |
| Valborg |  | Gudmundsdóttir |
| Ramneek |  | Gupta |
| Mark |  | Haid |
| Torben |  | Hansen |
| Tue | H. | Hansen |
| Andrew | T. | Hattersley |
| Ragna |  | Haussler |
| Alison | J. | Heggie |
| Anita | M. | Hennige |
| Anita | V. | Hill |
| Reinhard | W. | Holl |
| Michelle |  | Hudson |
| Bernd |  | Jablonka |
| Ulrik Plesner |  | Jacobsen |
| Christopher |  | Jennison |
| Joachim |  | Johansen |
| Angus | G. | Jones |
| Tugce |  | Karaderi |
| Jane |  | Kaye |
| Gwen |  | Kennedy |
| Maria |  | Klintenberg |
| Tarja |  | Kokkola |
| Anitra | D. | Koopman |
| Azra |  | Kurbasic |
| Teemu |  | Kuulasmaa |
| Markku |  | Laakso |
| Thorsten |  | Lehr |
| Heather |  | Loftus |
| Agnete Troen | T. | Lundgaard |
| Liwei |  | Lyu |
| Anubha |  | Mahajan |
| Andrea |  | Mari |
| Gianluca |  | Mazzoni |
| Mark | I. | McCarthy |
| Timothy | J. | McDonald |
| Donna |  | McEvoy |
| Nicky |  | McRobert |
| Ian |  | McVittie |
| Miranda |  | Mourby |
| Petra |  | Musholt |
| Pascal |  | Mutie |
| Rachel |  | Nice |
| Claudia |  | Nicolay |
| Giel |  | Nijpels |
| Birgitte |  | Nilsson |
| Colin | N. | Palmer |
| Francois |  | Pattou |
| Imre |  | Pavo |
| Helle | K. | Pedersen |
| Oluf |  | Pedersen |
| Mandy | H. | Perry |
| Hugo |  | Pomares-Millan |
| Cornelia | P. | Prehn |
| Anna |  | Ramisch |
| Simon |  | Rasmussen |
| Violeta |  | Raverdi |
| Martin |  | Ridderstråle |
| Neil |  | Robertson |
| Marianne |  | Rodriquez |
| Hartmut |  | Ruetten |
| Femke |  | Rutters |
| Peter |  | Sackett |
| Nina |  | Scherer |
| Nisha |  | Shah |
| Sapna |  | Sharma |
| Iryna |  | Sihinevich |
| Nadja | B. | Sondertoft |
| Hans-Henrik |  | Staerfeldt |
| Birgit |  | Steckel-Hamann |
| Harriet |  | Teare |
| Cecilia Engel | E. | Thomas |
| Elizabeth Louise | L. | Thomas |
| Melissa | K. | Thomas |
| Henrik | S. | Thomsen |
| Barbara |  | Thorand |
| Claire | E. | Thorne |
| Joachim |  | Tillner |
| Konstantinos | D. | Tsirigos |
| Andrea |  | Tura |
| Mathias |  | Uhlen |
| Sabine |  | van Oort |
| Jagadish |  | Vangipurapu |
| Helene |  | Verkindt |
| Henrik |  | Vestergaard |
| Ana |  | Viñuela |
| Josef | K | Vogt |
| Peter | W. | Wad Sackett |
| Mark |  | Walker |
| Agata |  | Wesolowska-Andersen |
| Brandon |  | Whitcher |
| Margaret | W. | White |
