# Supplementary material for: The influence of metformin treatment on the circulating proteome
Source: eBioMedicine. 2025 Jul 19;118:105859. doi: 10.1016/j.ebiom.2025.105859 (PMC12301841; doi:10.1016/j.ebiom.2025.105859)
Supplement: IMI-RHAPSODY Group Author List [file mmc4.docx]

| **First name** | **Middle initials** | **Surname** |
| --- | --- | --- |
| Alexander |  | Efanov |
| Giuseppe | N. | Giordano |
| Gerard | A. | Bouland |
| Frédéric |  | Burdet |
| Iulian |  | Dragan |
| Andreas |  | Festa |
| Michael | K. | Hansen |
| Dmitry |  | Kuznetsov |
| Florence |  | Mehl |
| Diana |  | Marek |
| Imre |  | Pavo |
| Kevin |  | Duffin |
| Samreen | K. | Syed |
| Janice | L. | Shaw |
| Over |  | Cabrera |
| Timothy | J. | Pullen |
| Bernard |  | Thorens |
| Mark |  | Ibberson |
| Guy | A. | Rutter |
